# The contribution of DNA methylation to the (dys)function of oligodendroglia in neurodegeneration

**DOI:** 10.1186/s40478-023-01607-9

**Published:** 2023-06-29

**Authors:** Katherine Fodder, Rohan de Silva, Thomas T. Warner, Conceição Bettencourt

**Affiliations:** 1 Queen Square Brain Bank for Neurological Disorders, UCL Queen Square Institute of Neurology, London, UK; 2 Department of Neurodegenerative Disease, UCL Queen Square Institute of Neurology, London, UK; 3 Department of Clinical and Movement Neurosciences, UCL Queen Square Institute of Neurology, London, UK; 4 Reta Lila Weston Institute, UCL Queen Square Institute of Neurology, London, UK

**Keywords:** DNA methylation, Oligodendrocytes, Neurodegeneration, Myelin, Epigenetics, Human brain

## Abstract

Neurodegenerative diseases encompass a heterogeneous group of conditions characterised by the progressive degeneration of the structure and function of the central or peripheral nervous systems. The pathogenic mechanisms underlying these diseases are not fully understood. However, a central feature consists of regional aggregation of proteins in the brain, such as the accumulation of β-amyloid plaques in Alzheimer’s disease (AD), inclusions of hyperphosphorylated microtubule-binding tau in AD and other tauopathies, or inclusions containing α-synuclein in Parkinson’s disease (PD), dementia with Lewy bodies (DLB) and multiple system atrophy (MSA). Various pathogenic mechanisms are thought to contribute to disease, and an increasing number of studies implicate dysfunction of oligodendrocytes (the myelin producing cells of the central nervous system) and myelin loss. Aberrant DNA methylation, the most widely studied epigenetic modification, has been associated with many neurodegenerative diseases, including AD, PD, DLB and MSA, and recent findings highlight aberrant DNA methylation in oligodendrocyte/myelin-related genes. Here we briefly review the evidence showing that changes to oligodendrocytes and myelin are key in neurodegeneration, and explore the relevance of DNA methylation in oligodendrocyte (dys)function. As DNA methylation is reversible, elucidating its involvement in pathogenic mechanisms of neurodegenerative diseases and in dysfunction of specific cell-types such as oligodendrocytes may bring opportunities for therapeutic interventions for these diseases.

## Introduction

Neurodegenerative diseases form a diverse group of neurological disorders which are characterised by progressive degeneration of the structure and function of the central or peripheral nervous system accompanied by loss of neurons. Despite the extensive accumulation of evidence and proposal of multiple pathogenic mechanisms, there is much to learn about how these disorders develop and progress. Many studies to date have focused on neuronal cells [33, 36]. Less is understood of the involvement of oligodendrocytes (OLGs), which are a major type of glial cells in the central nervous system (CNS). The primary function of OLGs is to produce myelin, and there is growing evidence which implicates myelin changes and OLG dysfunction across several neurodegenerative diseases [7, 14, 25, 77, 79, 87, 102]. In support of these changes not being mere downstream consequences of disease, multiple genome-wide association studies (GWAS) have identified genetic variants in myelin/OLG-related genes such as *MOBP* as being associated with the risk of multiple neurodegenerative diseases [18, 41, 46, 54, 59, 85, 88]. However, a more holistic view of mechanisms underlying OLG malfunction in neurodegeneration is far from being fully elucidated.

Changes to the epigenome have been consistently associated with neurodegenerative diseases in recent years [4, 73, 91, 99, 118]. By inducing chromatin changes, epigenetic mechanisms can regulate gene expression without changing the underlying genetic sequence. These epigenetic mechanisms include DNA methylation, which is the most widely studied, and histone modifications such as acetylation [73]. Although epigenetic modifications are crucial for the functioning of the cells and are involved in processes such as genomic imprinting and tissue differentiation, they also relate to the development of disease [67, 73]. Of interest to the topic to be discussed in this review, studies highlighting cell-type specific epigenetic changes [91], including in OLGs, started to emerge in the field of neurodegeneration. Although at its infancy, such studies are much needed to fully understand cell-type specific contributions to disease processes.

In this review, we highlight evidence which implicates the (dys)function of OLGs and myelin in neurodegeneration, and discuss how epigenetic modifications such as DNA methylation are crucial for OLG life cycle and myelination and how this could be affected in disease.

## The importance of the oligodendrocyte lineage and the myelin in the healthy brain

OLGs are a major glial cell type in the CNS, which constitute around 75% of the CNS glial cell population [80]. OLGs are responsible for the production, stability, and maintenance of myelin [14], the lipid-rich, multilamellar membrane which wraps around axons and enables fast transmission of electrical signals. Structurally, the myelin sheath is an extension of the OLG plasma membrane that wraps around nerve axons in a concentric fashion [102]. The myelin sheath is not continuous along the neuron. Sections of myelinated axon are separated by nodes of Ranvier. These enable saltatory conduction, the ‘hopping’ of electrical impulses along axons, which allows for fast transmission of electrical signals. The importance of the myelin sheath is demonstrated by the consequences of its loss, notably in demyelinating diseases such as multiple sclerosis (MS), where it results in a range of neurological symptoms including visual, motor and sensory problems, with associated disability and reduced life expectancy [20]. OLGs are also involved in homeostasis, trophic support to neurons, provision of lactate to neurons, and the secretion of various growth factors [26].

**Fig. 1:**
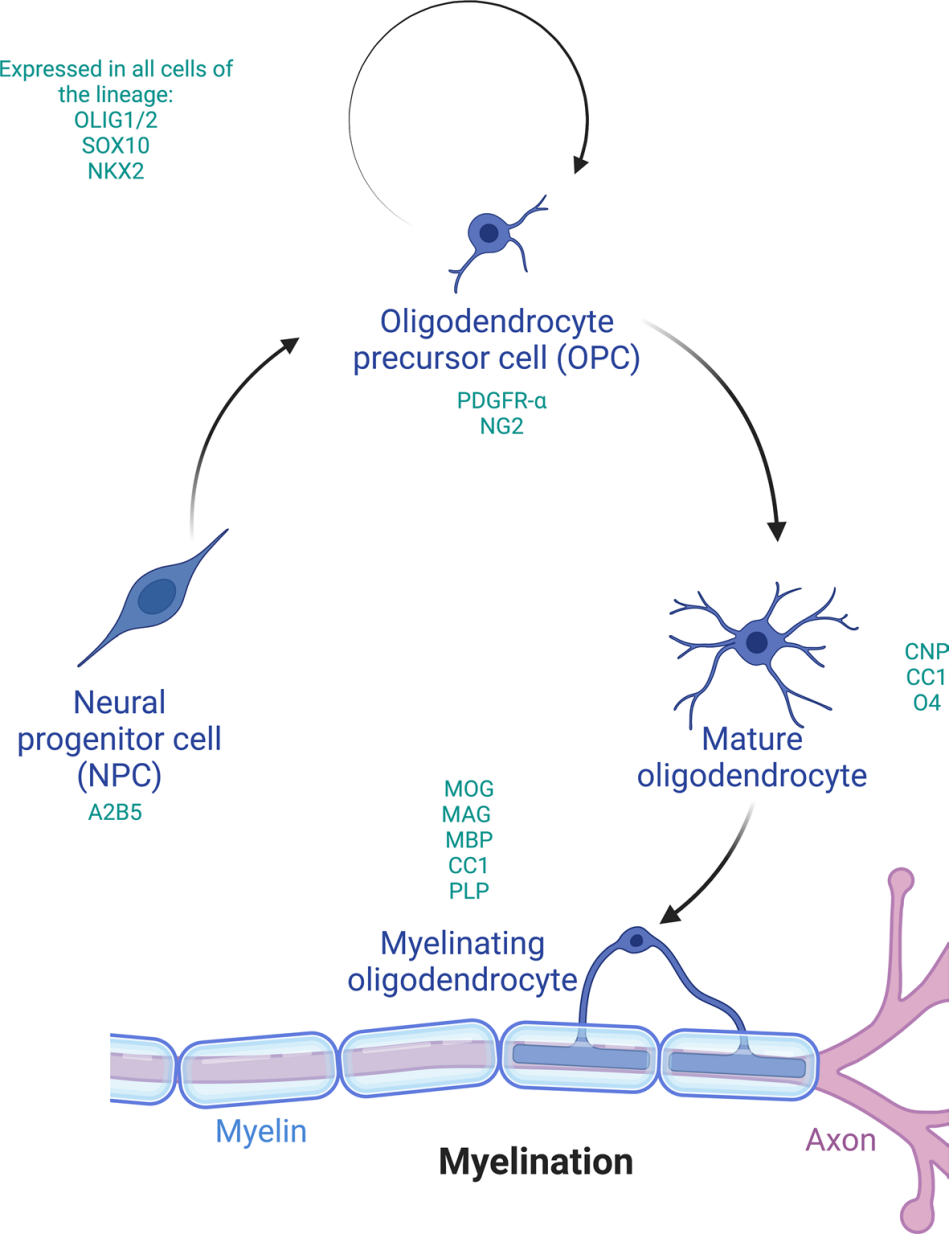
Schematic representation of the stages of OLG lineage differentiation. OPCs (PDGFRα^high^/NG2^+^) arise from NPCs (A2B5^+^), before forming mature OLGs (O4^+^/CNP^+^/CC1^+^) and then myelinating OLGs (MOG^+^/MAG^+^/MBP^+^/CC1^+^/PLP^+^). *NPC* Neural progenitor cell*, OLG* Oligodendrocyte, *OPC* Oligodendrocyte precursor cell. Figure created with BioRender

OLGs arise from oligodendrocyte precursor cells (OPCs), which are characterised by the expression of PDGFR-α (platelet derived growth factor receptor α) and NG2 (neuron-glial antigen 2) [107]. It is known that OPCs, which arise in the ventricular zone during early development [106, 108, 110], proliferate and migrate, and differentiate in stages [56] into myelinating OLGs (Fig. 1). Although most OPCs differentiate to form myelinating OLGs, some OPCs are retained in their proliferative stage. This results in OPCs accounting for 5–10% of all adult brain cells [22]. The main role of adult OPCs is to provide a source of new mature, myelinating OLGs. However, recent studies show they also have other important roles, including their involvement in cell signalling, metabolic regulation and as immune modulators [28, 52, 76]. The maturation of OPCs into OLGs, although relatively well characterised in mice, is not well described in humans given the technical challenges of studying post-mortem brain tissue and/or limitations of current human OLG-like cell lines.

## A role for myelin and oligodendrocytes in neurodegenerative diseases

### The involvement of myelin changes in neurodegeneration

**Fig. 2:**
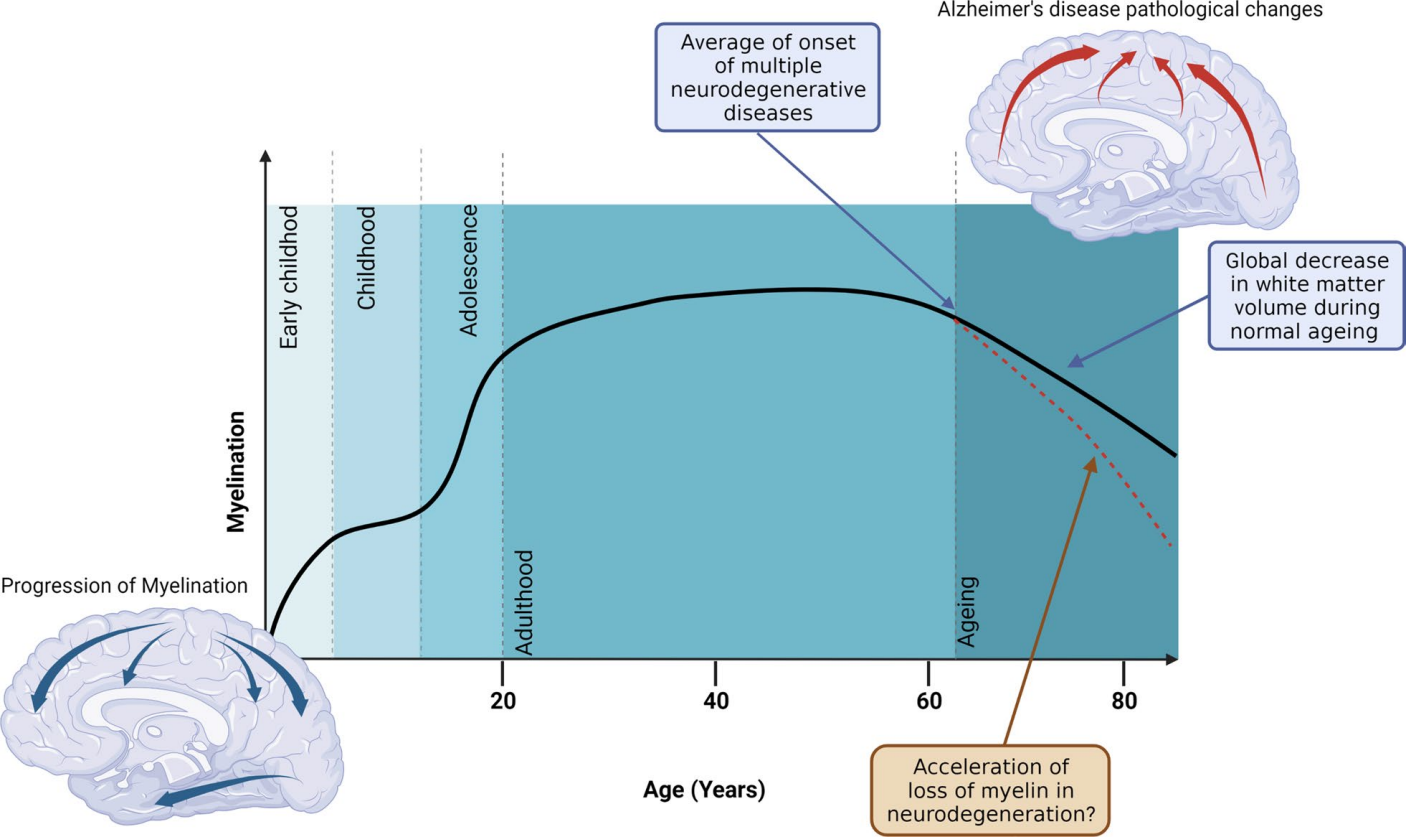
Schematic of the myelin changes throughout life. Shaded areas indicating myelination waves (as defined by de Faria et al. [29]). Also depicted are visualizations of the progression of cortical myelination and the progression of Alzheimer’s disease related destruction. The average age of onset of multiple neurodegenerative diseases is also indicated and coincides with the start of normal ageing-related decline in myelination, which is hypothesized to be accelerated in neurodegeneration. Figure created with BioRender

Myelination is a dynamic process that continues throughout life. Most myelination takes place from early childhood through to adolescence, with the major part taking place in the first two years of life. However, myelination does continue into adulthood, followed by an age-related decline in myelination occurring around the sixth decade of life (Fig. 2) [29]. However, decreases in myelin with ageing are not uniform, with regions of the brain that are myelinated earlier in development (such as the primary motor and sensory regions) undergoing white matter decline later [87].

Although neurodegenerative diseases such as Alzheimer’s disease (AD) are mainly associated with grey matter and neuronal damage, there is evidence for decline and involvement of white matter during disease progression. Disruption of myelin in AD was described at the beginning of the twentieth century by Alois Alzheimer [79]. It has also been noted that the typical age-of-onset of neurodegenerative diseases coincides with the time when age-related decline in myelination is observed (Fig. 2) [29]. Moreover, early evidence of the disruption of myelin in AD suggested those regions of the cortex, such as the temporal and frontal lobes, that are myelinated later in development are more likely to present with AD pathology earlier [13, 79]. This suggests that those regions that myelinate later are more vulnerable to pathogenic mechanisms which result in neurodegeneration. Further evidence of this involvement of myelin comes from the observations of white matter changes in brain imaging studies. For example, white matter hyperintensities (WMHs), which are associated with loss of myelin integrity, have been shown to predict incident AD [15, 16, 77]. Brain imaging data has indicated that β-amyloid deposition may change white matter microstructure in early disease stages [23]. White matter abnormalities and myelin degradation are also described in other neurodegenerative diseases, including multiple system atrophy (MSA) [64], amyotrophic lateral sclerosis (ALS) [121] and progressive supranuclear palsy (PSP) [27, 114].

### The involvement of the oligodendrocyte lineage in neurodegeneration

#### Evidence from pathology

A direct role for OLGs in neurodegenerative disease is exemplified by the pathology of MSA, where glial cytoplasmic inclusions (GCIs) in OLGs are the pathological hallmark of the disease [49]. In MSA, these inclusions consist of aggregates of the synaptic protein α-synuclein. Whether α-synuclein is produced by the OLGs or propagated from neurons is not clear. In MSA, increased number of OPCs is also reported in post-mortem brain tissue [1, 65]. PSP and corticobasal degeneration (CBD) also display clear OLG pathology with disease hallmarks including tau deposits in OLGs, presenting as coiled bodies [24, 53].

Although the precise role of OLGs in AD pathology is less clear, there is evidence from human post-mortem studies that there are alterations in the numbers and morphology of OLG lineage cells in this disease [77]. In post-mortem AD brain tissue, decreases in Olig2 + cells have been reported [8], as well as increased numbers of OPCs in white matter lesions [93]. Morphological changes in OLGs derived from AD post-mortem brains have also been seen, specifically a decrease in nuclear diameter in parahippocampal white matter [34]. A recent study, in apolipoprotein E-ε4 allele (*APOE*-ε4) carriers, also demonstrated aberrant deposition of cholesterol in OLGs and dysregulated myelination in AD [12].

#### Evidence from genetics

GWAS have also implicated specific myelin/OLG-related genes in neurodegeneration, including the bridging integrator 1 (*BIN1*) gene, which is the second strongest genetic risk factor for late onset AD [45, 57, 58] and known to be largely expressed by mature OLGs and localised to white matter tracts [86]. Increased expression of *BIN1* is reported in AD [17], although mechanisms behind the association of *BIN1* and AD are unclear. Myelin associated oligodendrocyte protein (*MOBP*) gene has been associated with disease risk in several neurodegenerative diseases, including PSP [19, 41, 88], CBD [54], AD *APOE*-ε4 carriers [59], ALS [85] and PD [95], and has also been reported to be associated with white matter degradation and increased rates of decline in executive function in behavioural variant frontotemporal dementia [46]. The functional repercussions of such associations remain unclear. However, in human brain tissue, the risk allele T, of the disease-associated single nucleotide polymorphism rs1768208, is also associated with increased expression of the *MOBP* gene in PSP [2]*.*

**Fig. 3:**
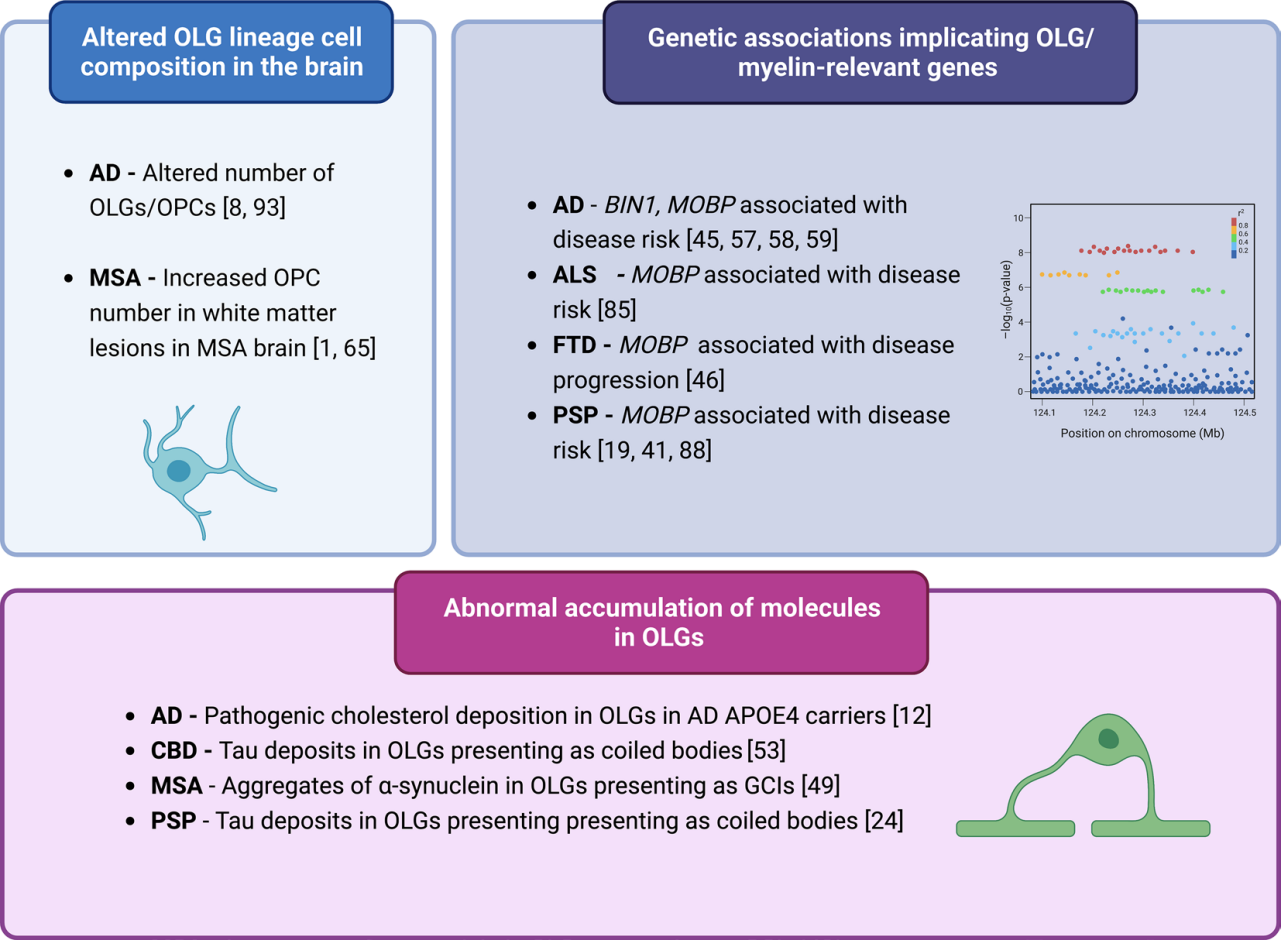
Non-exhaustive summary of evidence implicating the oligodendrocyte lineage across neurodegenerative diseases. *AD*—Alzheimer’s disease, *ALS*—Amyotrophic lateral sclerosis, *BIN1*—Bridging Integrator 1, *CBD*—Corticobasal degeneration, *FTD*—Frontotemporal dementia, *GCI* – Glial cytoplasmic inclusion, *MSA*—Multiple System Atrophy, *MOBP*—Myelin-associated oligodendrocyte protein, *OLG*—Oligodendrocyte, *OPC*—Oligodendrocyte precursor cell, *PSP*—Progressive supranuclear palsy. Figure created with BioRender

Aside from such examples of myelin/OLG relevant genes identified through GWAS, transcriptomic analyses reveal gene expression changes in additional myelin-related genes in a broad range of neurodegenerative diseases, including AD [3], PSP [3], MSA [82], and frontotemporal lobar degeneration (FTLD) [39], further supporting the idea of myelination changes as a common pathway across these diseases. Examples of evidence supporting the importance of OLG/OPC involvement across several neurodegenerative diseases are given in Fig. 3.

## A role for DNA methylation in the dysfunction of oligodendrocytes and myelin in neurodegenerative diseases

**Fig. 4:**
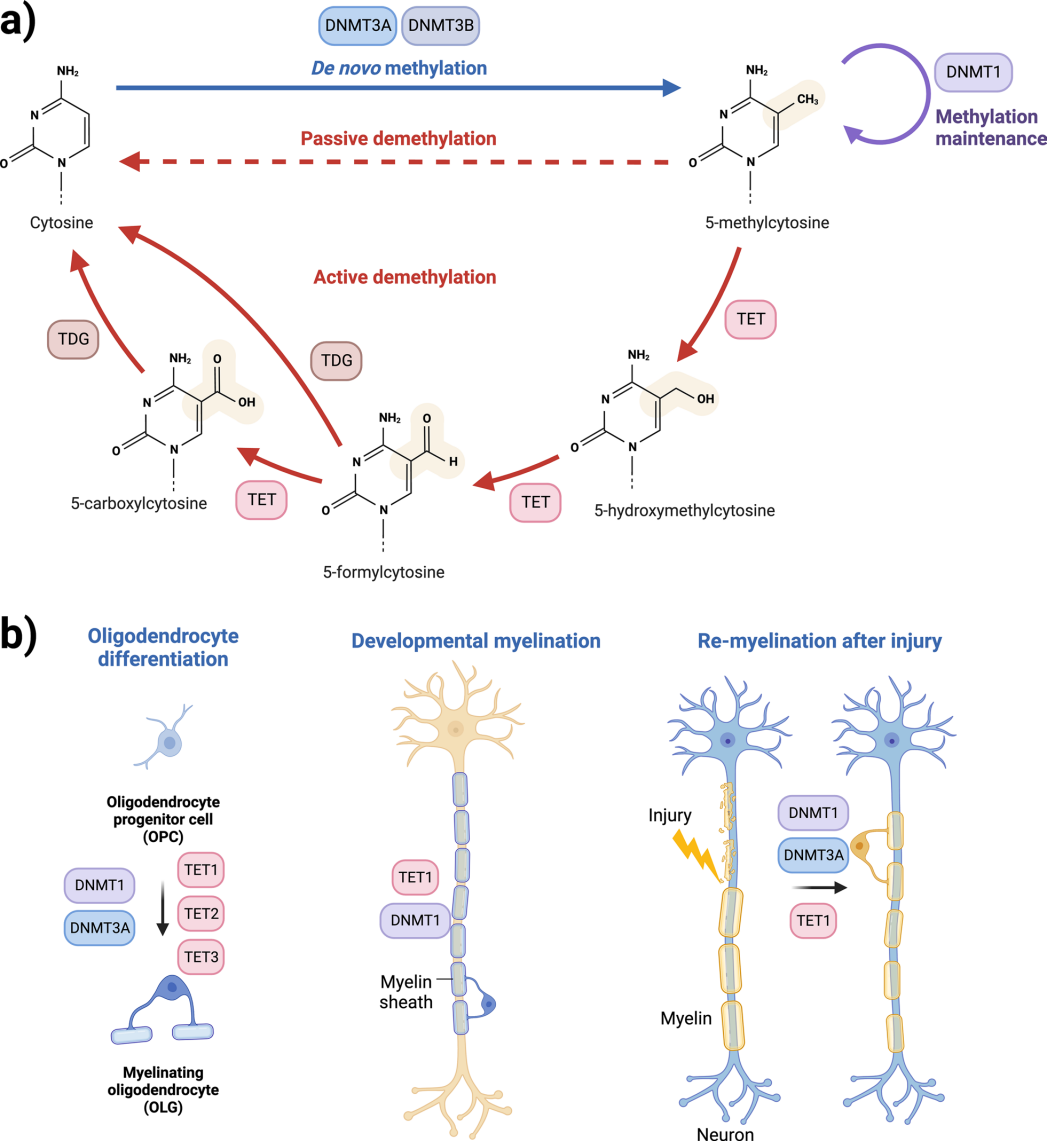
Schematic representation of the DNA modifications cycle, including factors responsible for the transitions between states. **a** Overall DNA modifications cycle; **b** Diagram summarising the known involvement of DNMTs and TET enzymes in OLG differentiation, developmental myelination, and in remyelination in response to injury. Evidence shows an age dependent role for DNMT1 and DNMT3A, with the former suggested to be more important in developmental myelination, and the latter in the remyelination involving differentiation of adult OPCs [68, 71]. Whilst it has been suggested that all three TET enzymes are involved in oligodendrocyte differentiation [120], TET1 has been reported to be more important for myelination and remyelination after injury [38, 69]. *DNMT1/3A/3B*—DNA methyltransferase 1/3A/3B, *TET*—ten-eleven translocation enzymes, *TDG*—thymine DNA glycosylase. Figure created with BioRender

**Fig. 5:**
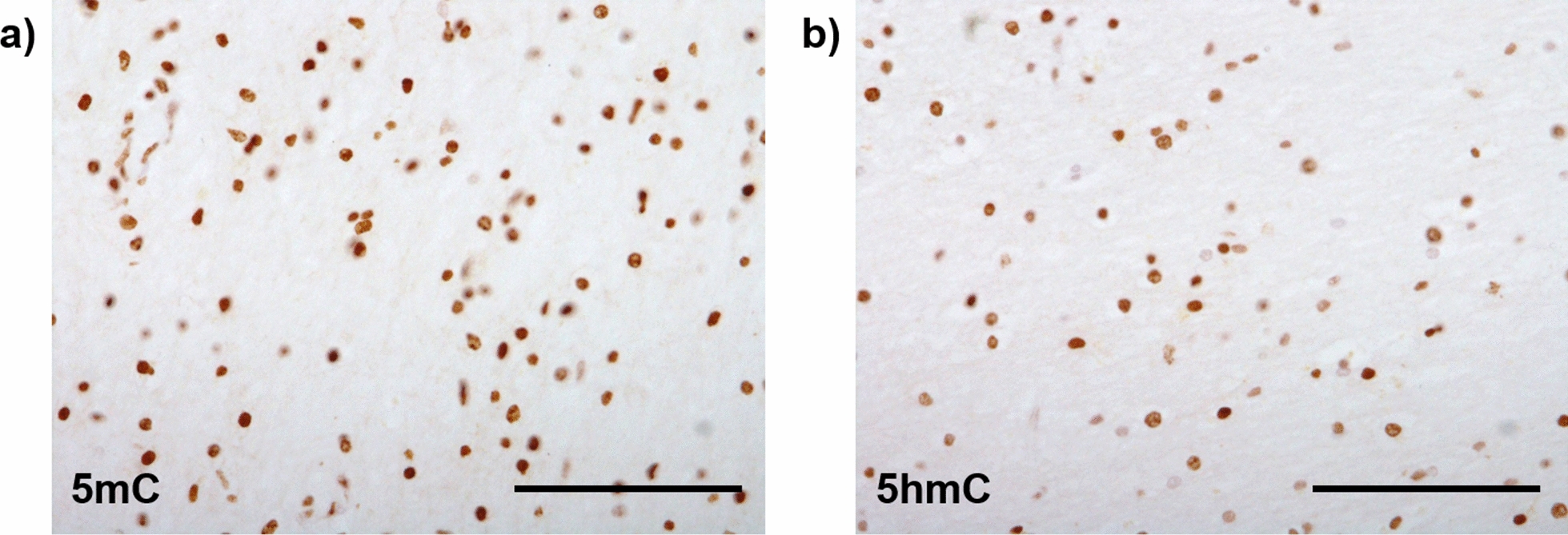
Immunohistochemical analysis of global DNA (hydroxyl)methylation in human post-mortem cerebellar white matter tissue. Positive staining in nuclei of glial cells, including oligodendrocytes, is shown in brown (scale bar 100 µm). **a** 5‐Methylcytosine (5mC) immunohistochemical staining; **b** 5‐Hydroxymethylcytosine (5hmC) immunohistochemical staining

Epigenetic modifications are those which, without altering the underlying DNA sequence, bring around changes in gene expression. DNA methylation, the most widely studied epigenetic modification, involves the transfer of a methyl group to a cytosine nucleotide to form 5-methylcytosine (5mC) [66] (Fig. 4a). This transfer is catalysed by a family of enzymes called DNA methyltransferases. The effect DNA methylation on gene expression regulation is largely dependent upon genomic location [37, 66]. For example, methylation within the gene body, i.e. protein coding exons and introns, more often results in increased gene expression, whereas methylation in the promoter region frequently leads to decreased gene expression [60, 78, 109]. DNA methylation, along with other epigenetic modifications, allow the intricate spatiotemporal control of gene expression and is crucial both during development and adult life. DNA methylation has been implicated in many processes relevant for the brain, including in brain development, learning, memory, and brain cell-type specification [48]. DNA hydroxymethylation (5hmC), an oxidative derivative of DNA methylation (Fig. 4a), is also important. Having originally been presumed to be an intermediate mark before demethylation [38], evidence now supports a functional role for 5hmC [103]. Interestingly, the distribution of different methylation states varies in a tissue dependent manner, with 5hmC known to be enriched tenfold in human brain compared to peripheral tissues [35]. Distribution of 5mC and 5hmC between brain cell-types has also been reported to be variable, with studies indicating that 5hmC may be enriched in neuronal cells compared to OLGs [55, 72]. Notwithstanding, Fig. 5 shows 5mC and 5hmC immunopositive glial nuclei, including OLG nuclei, in human post-mortem white matter tissue.

Studies using immunodetection of 5mC or 5hmC have often failed to lead to consensus regarding the occurrence of global DNA methylation/hydroxymethylation changes in neurodegenerative diseases, possibly reflecting limitations of such techniques [5, 73, 113]. However, technological advances that allowed querying throughout the genome at specific sites, have empowered investigations of relevant candidate genes and epigenome-wide association studies (EWAS) to identify DNA methylation alterations in neurodegenerative diseases at the single nucleotide resolution. In AD, EWAS studies utilising bulk brain tissue have identified multiple genes with DNA methylation changes associated with the disease and its pathological burden [47, 61, 98, 111], and meta-analyses have identified significant changes across multiple brain regions [91, 96, 99, 118]. Differentially methylated genes have also been identified in bulk brain tissue EWAS in other neurodegenerative diseases such as PD [51, 63], PSP [112], MSA [10], FTLD [31] and Huntington’s disease [43, 100]. DNA methylation changes in AD and movement disorders (including PD, HD, PSP and MSA) have been reviewed by Smith et al. [96] and Murthy et al. [73], respectively.

**Fig. 6:**
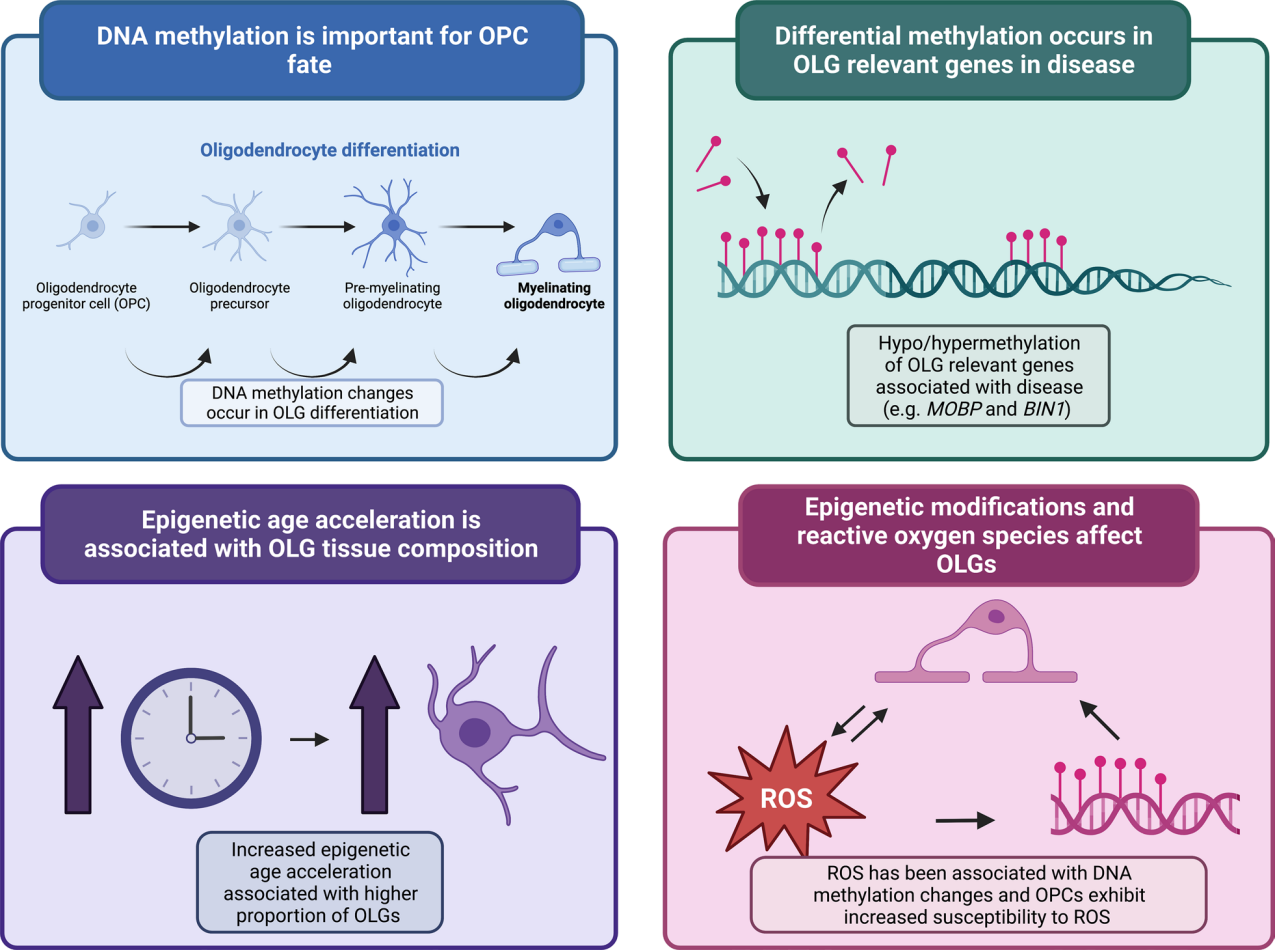
Potential roles for DNA methylation in the dysfunction of oligodendrocytes and myelin in neurodegenerative diseases. Panels illustrate different lines of evidence that implicate DNA methylation changes affecting OLGs/OLG-related genes and their relevance to neurodegeneration. *BIN1*—Bridging interactor 1, *MOBP*—Myelin associated oligodendrocyte protein, *OPC*—Oligodendrocyte precursor cell, *OLG*—Oligodendrocyte, *ROS*—Reactive oxygen species. Figure created with BioRender

Whilst most DNA methylation studies employ ‘bulk’ tissue analysis, more recent studies exploring DNA methylation changes in neurodegeneration have investigated cell-specific alterations. Shireby and co-workers investigated AD-related DNA methylation signatures in purified brain nuclei and found that many AD-related DNA methylation changes that had previously been detected in ‘bulk’ tissue were indeed driven by changes in non-neuronal cells, including in OLGs [91]. This finding highlights the need of a deeper understanding of DNA methylation changes in OLGs which may also occur in other neurodegenerative diseases. Below, we discuss several lines of evidence that support DNA methylation having a role in the dysfunction of OLGs and myelin in neurodegenerative diseases. These are summarised in Fig. 6.

### DNA methylation plays a crucial role in determining the fate of OPCs in health and neurodegenerative diseases

Gene expression changes determined by DNA methylation play an important role in the process of lineage specification from OPCs to mature OLGs [68, 105]. Although most studies investigating the OLG life cycle have been conducted in animal models, there is significant evidence to suggest that DNA methyltransferases and DNA methylation are dynamic in the processes of OPC specification, survival, proliferation, differentiation, and myelination [68, 70, 83, 104, 105]. Proliferation of OPCs occurs in response to exogenous signals such as growth factors, and epigenetic modifications are important players in this regulation. In mice, ablation of DNA methyltransferases has been shown to result in a hypomyelinating phenotype through reduction in the OPC progenitor pool due to impaired OPC proliferation [70]. During differentiation of OPCs to OLGs in mice, lower DNA methylation levels in myelin genes and increased methylation levels in cell cycle and neuronal genes were reported [70]*.* Given that the majority of the DNA methylation sites (CpGs) investigated in these studies were in promoter regions, and given the association of promoter region DNA hypermethylation with decreased gene expression, this supports an important role for DNA methylation in silencing cell cycle and proliferation genes and in activating myelin genes, thus enabling OPCs to leave their proliferative state and differentiate into myelinating OLGs. DNA methylation of specific genes involved in OPC differentiation has also been described. The DNA-binding protein inhibitors *Id2* and *Id4* showed decreased expression during OPC differentiation in mice, which was correlated with hypermethylation of their promoter regions, suggesting a role for DNA methylation in the silencing of these genes to allow the expression of myelin genes during differentiation [104]. As described in MSA and AD brain tissue, the increased number of OPCs observed [26, 65, 93] could be reflective of an inability of the OPCs to mature and differentiate into myelinating OLGs, possibly in part due to defective DNA methylation. However, further investigations are required to shed light on such effects. It is also worth noting that DNA methyltransferases DNMT1, DNMT3a and DNMT3b have been shown to have distinct roles in various aspect of the OLG lineage cell cycle, myelination, and in remyelination after injury (Fig. 4b) [68, 70, 71, 83, 105].

DNA hydroxymethylation is also dynamic during the OLG life cycle, and TET1 is one of the enzymes involved in this process (Fig. 4). Slower cell cycle progression of OPCs was found in *Tet1* knock-out mice, an effect that appeared to be largely specific to the OLG lineage compared to neurons and astrocytes [119]. TET1 was also found to be implicated in processes of myelin repair through the regulation of genes important for the axon-myelin interface [69]. Interestingly, there is increased hydroxymethylation in adult OPCs compared to neonatal OPCs in mice, and evidence suggests that TET1 is essential for myelin repair after damage [69]. As with DNMTs, there is evidence for distinct and complex roles of the TET enzyme family in different aspects of the OLG lineage cell life cycle (Fig. 4b) [69, 119, 120]. Overall, these studies indicate that DNA modifications undergo dynamic changes between neonatal and adult OPCs and are relevant for the decrease in myelinating capacity that is observed in ageing OPCs [87].

More research is needed to elucidate further the importance of 5mC and 5hmC in OLGs, and to understand the complex roles of DNMTs and the TET family of enzymes. This should include investigation of changes in their catalytic activities, during OLG differentiation, in myelination, and in remyelination after injury.

### Oligodendrocyte-related genes are differentially methylated in neurodegenerative diseases

As discussed above, OLG-related genes (e.g. *MOBP* and *BIN1*) have been associated in GWAS with the risk of developing neurodegenerative diseases. In addition, *MOBP* was shown to present aberrant DNA methylation in an EWAS of post-mortem MSA white matter tissue [10]. Specifically, hypermethylation (i.e. increased methylation levels) of the promoter region of the gene was detected in MSA compared to controls. DNA methylation changes in *MOBP* were found even in brain regions very mildly affected by MSA pathology (e.g. occipital lobe), indicating that these may reflect early changes and contribute to disease pathogenesis. The methylation status of *MOBP* in MSA is linked to changes in *MOBP* expression levels [11], and the observed downregulation of this gene is likely driven by the hypermethylation of its promoter region. As MOBP protein is involved in the morphological differentiation of OLGs [90], changes in its expression levels due to aberrant methylation during OLG differentiation likely lead to functional impairment of these cells. As another example, *BIN1* is the second strongest genetic risk factor for late onset AD [45, 57, 58] and associations between AD neuropathology and the level of methylation at the *BIN1* locus have also been reported [47, 116], with *BIN1* transcript levels being associated with β-amyloid load [116]. Given this, and that *BIN1* has been shown to be predominantly localised to white matter in the brain and expressed primarily in mature OLGs [86], it is reasonable to hypothesise that the involvement of *BIN1* gene in disease processes may be mediated through DNA methylation changes affecting OLGs.

### Oligodendrocyte cell types and epigenetic age acceleration

DNA methylation changes are known to occur during ageing, which is the major risk factor for neurodegeneration, with accelerated epigenetic ageing, as measured using epigenetic clocks, being reported in neurodegenerative diseases [42–44, 81]. Epigenetic clocks allow to infer the difference between the biological age, estimated using the DNA methylome, and the actual chronological age (i.e. epigenetic age acceleration). Age-related changes have been described in OLGs, notably the decrease in myelinating capacity with increased age [87], but it has also been suggested that there is a loss of epigenetic memory in these cells [92]. It has been proposed that intrinsic changes observed in ageing OLGs could be a result of changes on gene expression brought around by an altered epigenomic profile [92]. Indeed, a recent study investigating DNA methylation-based measures of accelerated ageing in post-mortem tissue from different brain regions in MSA and controls found that the relative frequency of OLGs in brain tissue is positively correlated with epigenetic age acceleration [75]. This relationship between OLG proportions and epigenetic age acceleration has also been found in some forms of FTLD [74]. These findings support a role for OLGs in pushing towards increased epigenetic/biological age, suggesting that this cell lineage ages faster than other brain cell types.

### Vulnerability of oligodendrocyte lineage cells to reactive oxygen species via epigenetic modifications

A further role of OLGs (aside from myelination) is their involvement in iron equilibrium in the CNS. Iron is key for normal CNS function [94], and OLGs are important in maintaining brain iron homeostasis [84]. Dysregulation, and, specifically, increased iron levels in the brain are associated with neurodegenerative diseases such as AD, PD, and MSA [117]. Proposed mechanisms for the role of iron in neurodegeneration include increased oxidative stress, possibly due to enhanced generation of reactive oxygen species (ROS) associated with increased protein aggregation [26, 117]. Given that OLGs are the principal iron-containing cells of the brain [21], it is reasonable to hypothesise that aberrant OLG function could contribute to neurodegeneration via dysregulation of brain iron levels. Indeed, investigations into brain gene expression in the context of neurodegeneration with brain iron accumulation (NBIA), and in mouse models of increased brain iron loading, implicate OLGs and myelin-related genes [9, 40]. This may well be relevant for other neurodegenerative diseases. OPCs and OLGs are thought to be more vulnerable to oxidative stress than other brain cell types due to factors which include lower levels of antioxidant enzymes and free radical scavengers [6, 30], as well as their high metabolic requirements [7, 87]. Excessive oxidative stress can lead to OLG malfunction through the impairment of effective OLG differentiation [32], with such effects having been reported in neurodegenerative diseases [101]. Interestingly, a link between DNA methylation changes and presence of ROS has been suggested with the finding that increased ROS leads to oxidation of 5mC into 5hmC [62], likely leading to changes in gene expression regulation. Although speculative, it could be hypothesised that this proposed increase in susceptibility of OPCs to ROS-induced damage compared to other brain cell types could, at least in part, be driven by ROS induced alterations in DNA methylation in these cells [6, 30, 32]. However, causal relationships between neurodegenerative processes, OPC dysfunction, DNA methylation and ROS are still unclear and require further investigations.

## Concluding remarks

There is an increasing understanding of the importance of myelin and OLGs in the pathogenesis of various neurodegenerative diseases, both in those with or without obvious OLG pathology. There is mounting evidence showing that the efficient development, proliferation, differentiation, and maintenance of the OLG lineage cells may be disrupted in disease, and that aberrant DNA methylation is implicated (Fig. 6). Whether such disease associated disruption leads to death of the OLG lineage cells and consequent demyelination, a decrease in their ability to provide trophic support to neurons, or another unknown ‘effect’ is still unclear. Thus, a better understanding of how human OLG lineage cells function in health and disease is needed. Currently, most studies of the role of epigenetic mechanisms in OLG function, including proliferation, have been carried out in animal models, with only limited studies in human OPCs/OLGs. It is also worth noting that other factors affecting gene expression, not within the scope of this review, including histone modifications and RNA methylation, may also be important in the life cycle of OLG lineage cells [83, 105, 115], and it is likely that combined effects from such factors contribute to OLG function both in health and disease.

Although EWAS have uncovered several aberrantly methylated genes across multiple neurodegenerative diseases, most of these studies utilised bulk tissue, limiting cell-type specific inferences. However, exciting recent advances in cell-type deconvolution algorithms and techniques allowing characterisation of DNA methylation changes in purified cell types or even in single cells will no doubt enhance the discovery of cell-type specific disease-associated DNA methylation signatures.

The study of DNA methylation in neurodegenerative diseases is an exciting avenue. On one hand, elucidating pathogenic mechanisms in disease could provide targets for therapeutic intervention. On the other hand, given that DNA methylation is potentially modifiable, and novel techniques to edit DNA methylation in specific genomic sites are emerging [50, 73, 89, 97], this reinforces the importance of knowing where in genome and in which cell-types disease-related changes occur, and could open new avenues for therapies targeting DNA methylation.
